# From Multi-omics To Personalized Training: The Rise of Enduromics and Resistomics

**DOI:** 10.1186/s40798-025-00855-4

**Published:** 2025-05-14

**Authors:** Kayvan Khoramipour, Sergio Maroto-Izquierdo, Simone Lista, Alejandro Santos-Lozano, Katsuhiko Suzuki

**Affiliations:** 1 i+HeALTH Strategic Research Group, Department of Health Sciences, Miguel de Cervantes European University (UEMC), Valladolid, 47012 Spain; 2 Faculty of Sport Sciences, Waseda University, Tokorozawa, Japan

## Abstract

Because of its positive effects on the cardiovascular, metabolic and neurohormonal systems, as well as other aspects of systemic physiology, exercise is crucial to overall health. Traditional exercise physiology techniques that rely on invasive procedures have limited our understanding of the molecular changes induced by exercise. This paper distinguishes the emerging fields of “*enduromics*” and “*resistomics*” from sportomics. *Enduromics and resistomics* concentrate on the molecular responses to endurance and resistance training, respectively, in a variety of populations, whereas sportomics stresses the study of molecular alterations in athletes in competitive or simulated situations. These fields integrate biological systems with omics technology to provide accurate insights into the many physiological responses that occur during aerobic and anaerobic exercise. These methods make it possible to create individualized training plans that maximise health, reduce injury risk and improve adherence by identifying biomarkers and metabolic fingerprints. The revolutionary potential of *enduromics and resistomics* for athletic performance and public health underscores the need for more research across all demographics and training modalities.

## Key Points

•. *Enduromics and resistomics*, as opposed to sportomics, which focusses on molecular alterations in competitive athletes, examine unique molecular adaptations to endurance and resistance training in a larger population.

• *Enduromics* uses multi-omics to reveal the molecular pathways that underpin aerobic efficiency, lipid metabolism, and mitochondrial biogenesis, providing complete insights into endurance training adaptations. *Resistomics*, on the other hand, uses multi-omics to investigate the molecular mechanisms that drive muscle hypertrophy, protein synthesis, and neuromuscular adaptations, resulting in a comprehensive knowledge of the impacts of resistance training.

• By utilizing molecular profiling, these disciplines offer personalized exercise prescriptions, which enhance results in metabolic health, fitness, and rehabilitation for clinical and non-clinical populations.

## Introduction

Exercise represents one of the foundations of human health promotion, benefiting cognition, immune and neurohormonal networks, as well as musculoskeletal and cardiorespiratory systems. Exercise, therefore, has beneficial effects spreading beyond individual organs and have a positive impact on systemic health [1–3].

Traditionally, the collection of in-depth molecular data in exercise physiology requires tissue or muscle biopsies [4], an invasive method that limits both the number of participants and the size of samples to be analyzed, thus reducing the measurable analytes. According to current estimates, there are more than 110,000 different metabolites in the human body and more than 46,000 metabolic signaling pathways [4]. Considering that most studies on exercise metabolism/physiology measure less than a dozen metabolites and examine only one to two metabolic pathways at a time, it is clear that only a tiny fraction of what can be measured is currently being studied [4].This scenario called for the development of global, unbiased, and less invasive approaches identifying all exercise-induced alterations in all tissues and metabolic pathways [4]. Indeed, since exercise induces significant physiological variations in several organs and tissues, the clear understanding of such alterations affecting large volumes of molecules (and, consequently, their associated pathways) is crucial [5]. In this regard, the emerging omics science, positioned in the systems biology (SB) framework, allows for comprehensive, unbiased, high-throughput, and minimally invasive molecular analyses in the field of exercise physiology.

The theoretical and computational framework of SB aims to understand biological entities at a systemic level, as it explores them not only as individual components, but also as interacting systems and examines their properties [6]. Hence, SB uses a holistic and integrative approach that allows for the simultaneous examination of sets of molecules (and their interactions), over time, at a network-system level [7].

The gradual integration of SB sciences into sports science research allowed the development of the paradigm of “*sportomics*”, denoting the use of integrative multi-omics sciences, in conjunction with traditional clinical medicine laboratory analyses (such as immunochemical-based assays, western blotting, and other analytical methods), to explore the molecular effects of sport induced alterations on athletes [8]. In general, a sportomics-based strategy simulates the real context encountered during competitions or simulated matches [9].

While exercise has traditionally been emphasized to enhance athletic performance, its impact on public health goes far beyond sport achievements. Physical activity is increasingly recognized as an effective preventive and therapeutic tool against non-communicable diseases (NCDs) such as diabetes, cardiovascular disease, and obesity, which together account for over 70% annual deaths worldwide [10]. According to the World Health Organization (WHO)’s Global Status Report on Physical Activity 2022, physical inactivity is expected to lead to nearly 500 million preventable NCDs cases by 2030, and contribute to an estimated $300 billion in global healthcare expenditure [11]. By promoting regular exercise as a health intervention and not just as a performance enhancer, we can reduce the financial burden on healthcare systems. This shift in focus, from elite sport to public health, highlights the broader, transformative potential of exercise to enhance societal health outcomes. Furthermore, if precision medicine continues to advance and is integrated into healthcare systems, individuals may indirectly benefit from these developments.

**Fig. 1:**
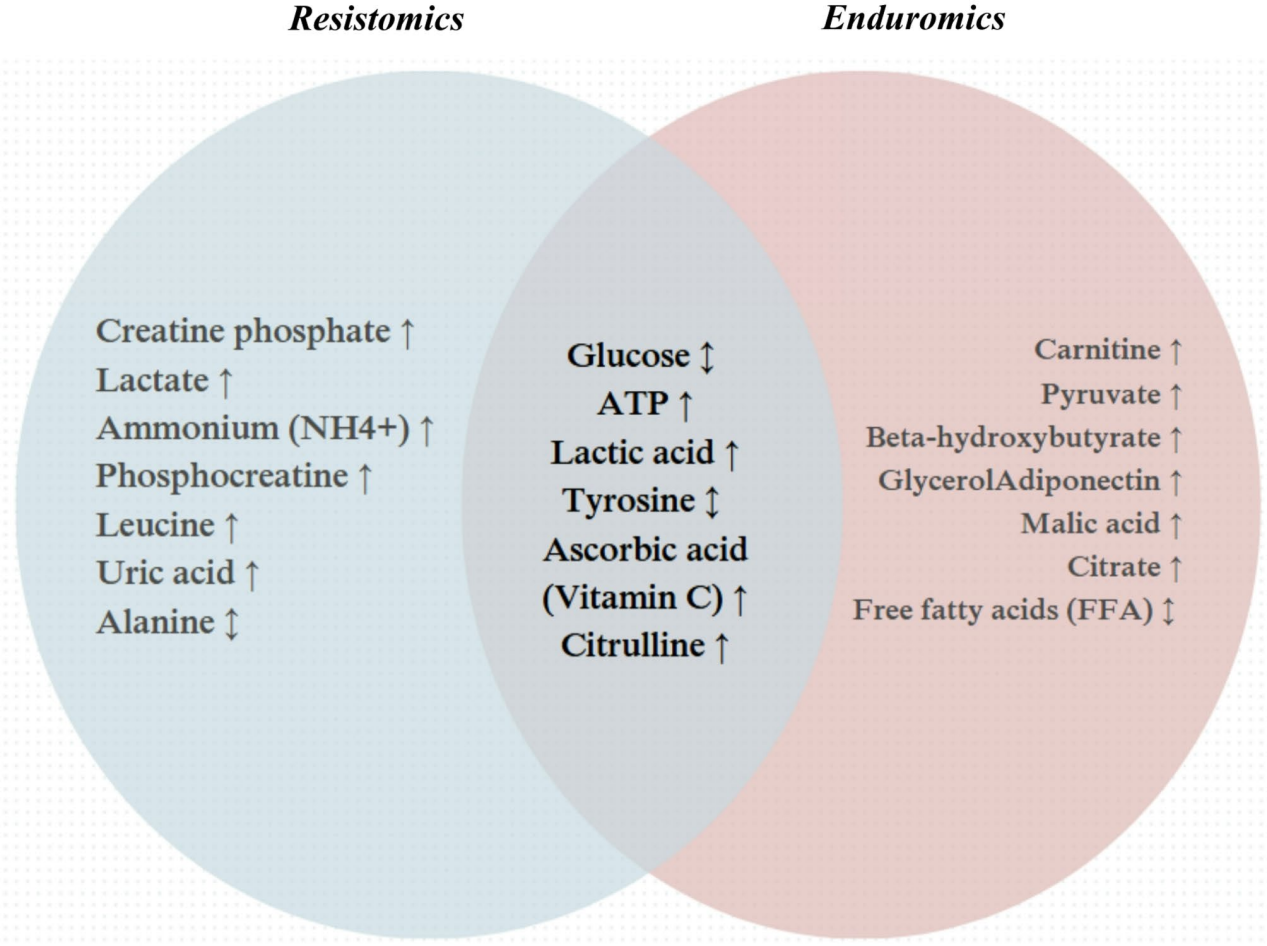
Potential metabolic alterations identified through *resistomics* (blue), *enduromics* (red), or both, based on data from Jaguri et al. (2023) and Morville et al. (2020), along with fundamental principles of exercise physiology [15, 16]. ↑Increase.↓Decrease. ↕ Load/context-dependent

Endurance and resistance training induce different metabolic and molecular adaptations that are crucial for different aspects of fitness [12]. Endurance training primarily targets mitochondrial biogenesis and increases oxidative capacity and fat oxidation [13]. On the other hand, resistance training induces muscle hypertrophy and strength gain [14]. Terms such as ‘*enduromics*’ (in relation to aerobic training) and ‘*resistomics’* (in relation to resistance training) may accurately capture the different responses and molecular mechanisms that account for the unique benefits of each exercise modality. Understanding these differences is essential for optimizing training strategies for different populations (Fig. 1).

## Proposal for *Enduromics* and *Resistomics*

*Enduromics* and *resistomics* build on the foundations of sportomics, but are more specific and attempt to analyze the molecular adaptations induced by *resistance and endurance* training. We define *enduromics and resistomics* as emerging fields that integrate various omics sciences (genomics/epigenomics, transcriptomics, proteomics, metabolomics/lipidomics) to study the molecular adaptations of endurance and resistance training. These fields aim to map the endurome and resistome, which represent the full spectrum of biological changes associated with these exercise modalities. These new concepts examine exercise-induced adaptations primarily in biofluids, including blood (plasma/serum), urine, and extracellular vesicles, to capture a more dynamic and holistic view of exercise adaptations.

*Enduromics* is the omics study of changes brought on by extended, moderate-to-intense aerobic exercise with the goal of finding biomarkers connected to enhanced mitochondrial biogenesis, lipid metabolism, and cardiorespiratory efficiency. *Resistomics*, on the other hand, focusses on looking at the molecular changes induced by resistance training, specifically how it affects protein synthesis, muscle damage [17], muscle hypertrophy [18], and neuromuscular adaptations [19], with an emphasis on identifying molecular markers specific to these processes (Fig. 1).

To gain a more thorough knowledge of how each training modality affects different health outcomes, such as muscular strength or cardiorespiratory fitness, and metabolic health, these -omics research should incorporate health-related fitness evaluations.

## Potential Applications of *Enduromics* and *Resistomics*

*Enduromics* and *resistomics* provide a transformative approach to personalized exercise prescriptions by using molecular insights to tailor training to individual profiles. Traditional training programs often apply generalized regimens without accounting for unique genetic and metabolic differences, whereas *enduromics* and *resistomics* offer a more targeted method, identifying specific biomarkers and physiological signatures associated with endurance or resistance adaptations. This precision enables improved exercise efficiency for both clinical and non-clinical populations, and promotes better outcomes in metabolic health, rehabilitation, and fitness.

*Enduromics* and *resistomics* studies may suggest potential molecular changes for each type of training. Combining this data with the initial profile of the participants, we can design more effective training programs [20]. For example, endurance exercise leads to coordinated molecular changes in inflammation, oxidative stress, energy metabolism and tissue healing, as shown in a longitudinal multi-omics analysis of blood components by Contrepois et al. [21]. These responses were particularly influenced by insulin resistance and biomarkers for peak oxygen consumption (VO_2peak_), a crucial fitness parameter, were discovered. Furthermore, a comparison of plasma metabolomics between resistance and endurance training was provided by Morville et al. [22]. They discovered that endurance training increased lipid oxidation and prolonged the upregulation of metabolites, while resistance training mainly altered nucleotide turnover and amino acid metabolism. The comprehensive analysis of plasma proteome adaptations to endurance training by Robbins et al. [23] identified hundreds of altered proteins associated with angiogenesis, extracellular matrix remodeling and oxygen uptake efficiency. They also linked these changes to improved cardiorespiratory fitness. All in all, researchers highlighted that the different omic responses to different forms of exercise that improve adaptations to resistance and endurance training, and emphasize the need to integrate multi-omics into tailored training plans.

This tailored approach not only promotes motivation and stamina by reducing training plateaus and improving results, but also supports sustainable, long-term fitness and wellness goals. In addition, training recommendations can be adjusted to match individual tolerance and recovery rates, minimizing the risk of injury and fostering sustained commitment.

Despite the promise of omics-based personalized exercise prescriptions, a major challenge remains: translating this knowledge into real-world behavioral changes. The integration of real-time omics data into wearable technologies would further advance this field, allowing for adaptive feedback that enables on-the-spot training adjustments based on immediate physiological responses [24]. Future developments in wearable technologies could facilitate “adaptive micro-cycles,” where training decisions are continuously refined in response to real-time molecular feedback. This level of precision training would help avoid cumulative fatigue, optimize the timing of nutrient delivery, and tailor recovery protocols to individual needs.

## The Future of Omics: Single-cell Multi-omics

A growingly relevant field of multi-omics is represented by single-cell multi-omics, accounting for individual cell type heterogeneity and features with unprecedented resolution by combining single-cell assays to concurrently profile multiple molecular layers in the same cell [25]. As a result, single-cell multi-omics allow exploring not only the genotypic and phenotypic traits of a definite cell, but also the integrated regulatory mechanisms that is possible to detect only at single-cell resolution [26]. This field is promising thanks to the continuous advancement of high-throughput methods, supported by powerful computational tools, facilitating the analysis in parallel of several omics layers, i.e. the exploration of various categories of molecules. This makes it feasible for the screening of thousands of individual cells all at once [27].

Single-cell multi-omics are increasingly applied to the field of exercise research, particularly to shed light on the impact of exercise on human phenotypes. They are expected to help disclose how exercise can induce physiological adaptations [28]. Moreover, given the progressive decrease in analyses costs, single-cell multi-omics will become more accessible. The implementation of advanced bioinformatics and computational biology tools will accelerate the practical use of all omics disciplines in exercise investigations [29].

Among all single-cell omics, single-cell metabolomics aids to enrich the advances achieved in single-cell analysis by genomics and proteomics; in particular, the aim is accurately charting and quantifying the metabolome to attain valuable information on cell function, with spatial resolution at the individual cell level [30]. Furthermore, as cellular metabolic pathways are dynamic and influenced by internal and external factors (differently from genome, transcriptome, and proteome), single-cell metabolomics can offer the most subtle dynamic picture to elucidate cell functions [31].

## Conclusion

*Enduromics and resistomics* are potential approaches to improve personalized training by using molecular insights to optimize training techniques. These developing disciplines of exercise science have the potential to improve not just sports performance, but also public health outcomes by providing personalized, evidence-based recommendations based on everyone’s unique physiology. This could also increase adherence, minimize risk of injury, and promote long-term health results. Future research should look at different demographic groups (e.g., ageing, chronic disease groups) and exercise protocols (e.g., training intensity, frequency, and type) to broaden the applicability of these concepts and improve our understanding of personalized exercise science and its role in preventive healthcare.

## Data Availability

Not applicable.

## List of Abbreviations

SB: Systems biology
NCDs: Non-communicable diseases
WHO: World Health Organization
VO2peak: Peak oxygen consumption
